# Asthma and Its Impact on Pediatric Patients Undergoing Surgical Management of Tibial Shaft Fractures

**DOI:** 10.7759/cureus.31369

**Published:** 2022-11-11

**Authors:** Meghan Tveit, Theodore Quan, Denver Kraft, Alisa Malyavko, Melina Recarey, Jordan Pizzarro, Chelsea Nguyen, Avilash Das, Pradip Ramamurti, Sean Tabaie

**Affiliations:** 1 Orthopaedic Surgery, George Washington University School of Medicine and Health Sciences, Washington DC, USA; 2 Orthopaedic Surgery, Georgetown University School of Medicine, Washington DC, USA; 3 Orthopaedic Surgery, University of Virginia School of Medicine, Charlottesville, USA; 4 Orthopaedic Surgery, Children's National Hospital, Washington DC, USA

**Keywords:** pediatrics, complications, asthma, treatment, tibial shaft fracture

## Abstract

Introduction

Tibial shaft fractures are a common presenting injury among the pediatric population. Asthma is also a common diagnosis that is frequently encountered in this population and has a significant impact on healthcare utilization, quality of life, and mortality. Given the high prevalence of these conditions and risks of peri-anesthetic respiratory complications, the purpose of this study was to evaluate an association between asthma and the incidence of 30-day postoperative complications following the surgical management of tibial shaft fractures in the pediatric population.

Methods

The National Surgical Quality Improvement Program-Pediatric database was used to identify pediatric patients who underwent surgical treatment for tibial shaft fractures from 2013-2019. Patients were categorized into two groups: patients with a history of asthma and patients without a history of asthma. Differences in patient demographics, comorbidities, and postoperative complications were assessed using bivariate and multivariate analyses.

Results

Of the 2,649 patients who underwent surgical treatment for tibial shaft fractures, 115 (4.3%) had asthma. Compared to those without asthma, patients with a history of asthma were more likely to have other medical comorbidities. After controlling for the differences in baseline characteristics between the two groups, patients with asthma had an increased risk of prolonged hospital stay (OR 5.78; 95% CI 1.67 to 20.00; p=0.006).

Conclusion

Pediatric patients being surgically treated for tibial shaft fractures with asthma had an increased risk of prolonged hospital stay. It is important that proper preoperative workup, perioperative care, and understanding of the implications of asthma on postoperative recovery are appreciated to reduce prolonged hospitalization lengths and minimize hospital costs associated with tibial shaft fracture surgery.

## Introduction

Among the pediatric population, tibial shaft fractures have been identified as the third most common long bone fracture [1]. Of these fractures, approximately 39% are located in the midshaft, 50% in the distal third, and 11% in the proximal third [1]. Young children and toddlers are at risk for tibial shaft fractures, even when the force of injury is low, and this injury pattern should be suspected in all children with a limp or refusal to bear weight [2]. Although most tibia fractures in children can be treated with casting, surgical intervention is considered for fractures that are open, irreducible, have failed nonoperative management, are associated with compartment syndrome, or are complicated by other life-threatening or limb-threatening injuries [2,3]. Surgical treatment options include open reduction and fixation with plates and screws, intramedullary nails, interlocking nails, or flexible nails depending on the fracture morphology and patient characteristics [3,4]. Across all therapeutic directions, the majority of pediatric tibial shaft fractures have excellent outcomes with patients returning to full activities [5].

Aside from pediatric injuries, asthma is one of the most prevalent conditions affecting children around the world [6]. Genetic susceptibility to asthma has been studied using genome-wide association studies and is considered an important risk factor for a child’s development of asthma [7]. However, the rapid increase in the prevalence of asthma and allergies globally has shifted the focus toward potential environmental and societal factors that contribute to the development of childhood asthma [8,9]. Environmental factors including air pollution and urbanization as well as family factors, including smoking, socioeconomic status, and parental income, have been associated with pediatric asthma [9]. The development of asthma is likely to result from a complex interplay between environmental, societal, and genetic factors.

Patients who are diagnosed with asthma inherently carry a risk of perioperative bronchospasm if undergoing procedures requiring the administration of anesthesia [10,11]. As asthma is a prevalent pediatric diagnosis, understanding how this diagnosis impacts outcomes following surgical procedures for common pediatric injuries is crucial to providing high-quality pediatric medical care. Therefore, the purpose of this study was to investigate whether an association exists between asthma and 30-day postoperative complications in pediatric patients undergoing surgical treatment for tibial shaft fractures.

## Materials and methods

The American College of Surgeons National Surgical Quality Improvement Program-Pediatric (ACS NSQIP-P) database was queried from 2013-2019. This database collects de-identified patient information at participating centers nationwide [12]. Current procedural terminology (CPT) codes 27756, 27758, and 27759 were used to identify all patients with tibial shaft fractures. Patients older than 18 years at the time of surgery were excluded. Patients missing baseline demographics data were also excluded. Patients were categorized based on a documented history of asthma including a history of asthma resulting in functional disability in daily activities, chronic medication requirement, or hospitalization for treatment of asthma.

Patient characteristics

Patient information, including age, gender, race, and the American Society of Anesthesiologists (ASA) class, were retrieved from the database. Comorbidities were collected, including cardiac (any cardiac risk factors, history of cardiac surgery, cardiopulmonary resuscitation within 7 days of surgery, or inotropic support at the time of surgery), renal (renal insufficiency or failure), neurological (seizure disorder, impaired cognitive status, structural central nervous system abnormality, cerebral palsy, or neuromuscular disorder), gastrointestinal (esophageal, gastric, or intestinal disease), and biliary (biliary, liver, or pancreatic disease) comorbidities. Steroid use within 30 days of surgery, nutritional support, bleeding or hematologic disorder, prior operation within 30 days, and preoperative blood transfusion were also recorded.

Postoperative outcomes

The 30-day postoperative complications assessed in this study included surgical site infections, wound dehiscence, pneumonia, urinary tract infection, bleeding requiring transfusion, venous thromboembolism, sepsis, extended length of hospital stay, return to the operating room, readmission, and mortality. Prolonged hospital stay was defined as > 5 days or one standard deviation above the mean length of stay for the patients in this study.

Statistical analysis

Pearson’s chi-squared test and analysis of variance were used to compare the differences in demographics, comorbidities, and postoperative outcomes between the two cohorts: patients with asthma and those without. To control for the differences in baseline characteristics, these covariates were included in the multivariate analysis for p-values < 0.20. Odds ratios with 95% confidence intervals were reported for the multivariate analysis results. A p-value of < 0.05 was considered to be statistically significant. IBM SPSS (Version 28; IBM Corp., Armonk, NY) was used for the analyses.

## Results

Characteristics

In total, 2,649 pediatric patients underwent treatment for tibial shaft fractures and were included in the study. Of these, 2,534 patients (95.7%) did not have a history of asthma while 115 (4.3%) had a history of asthma. Compared to those without asthma, patients with a history of asthma were younger (12.9 yrs vs 13.6 yrs, p=0.020) (Table 1). There was no difference in race or sex between the two groups (Table 1). Patients with asthma were more likely to have other medical comorbidities, including neurological (9.6% vs 3.5%; p<0.001), gastrointestinal (7.8% vs 1.1%; p<0.001), recent steroid use (1.7% vs 0.1%; p<0.001), nutritional support requirement (1.7% vs 0.4%; p=0.024), bleeding disorders (6.9% vs 0.0%; p<0.001), and hematologic disorders (2.6% vs 0.4%; p=0.002) (Table 2).

**Table 1:** Demographics and Clinical Characteristics of Patients Undergoing Surgical Treatment of a Tibial Shaft Fracture ¶Pearson’s chi-squared test **Analysis of variance Bolding equals significance p<0.05 ASA, American Society of Anesthesiologists; SD, standard deviation.

Demographics	No Asthma	Asthma	P-value
Total patients, n	2,534	115	
Sex, n (%)			0.962^¶^
Female	656 (25.9)	30 (26.1)	
Male	1,878 (74.1)	85 (73.9)	
Race, n (%)			0.525^¶^
White	1,555 (67.1)	71 (63.4)	
Black	404 (17.4)	27 (24.1)	
Hispanic	287 (12.4)	11 (9.8)	
American Indian	7 (0.3)	0 (0.0)	
Asian	57 (2.5)	3 (2.7)	
Native Hawaiian	7 (0.3)	0 (0.0)	
ASA, n (%)			< 0.001^¶^
I	1,153 (60.1)	13 (13.5)	
II	655 (34.2)	76 (79.2)	
III	107 (5.6)	7 (7.3)	
IV	3 (0.2)	0 (0.0)	
Mean age, yrs (SD)	13.59 (3.19)	12.88 (3.48)	0.020**

**Table 2:** Comorbidities of Patients Undergoing Surgical Treatment of a Tibial Shaft Fracture ¶Pearson’s chi-squared test Bolding equals significance p<0.05

Comorbidities	No Asthma	Asthma	P-value^ ¶^
Total patients, n	2,534	115	
Cardiac disease, n (%)	34 (1.3)	1 (0.9)	0.665
Renal disease, n (%)	0 (0.0)	0 (0.0)	-
Neurological disease, n (%)	89 (3.5)	11 (9.6)	< 0.001
Gastrointestinal disease, n (%)	29 (1.1)	9 (7.8)	< 0.001
Biliary disease, n (%)	2 (0.4)	0 (0.0)	0.719
Steroid use, n (%)	3 (0.1)	2 (1.7)	< 0.001
Nutritional support, n (%)	9 (0.4)	2 (1.7)	0.024
Bleeding disorder, n (%)	0 (0.0)	2 (6.9)	< 0.001
Hematologic disorder, n (%)	11 (0.4)	3 (2.6)	0.002
Prior operation within 30 days, n (%)	2 (1.2)	0 (0.0)	0.677
Preoperative blood transfusion, n (%)	2 (0.1)	0 (0.0)	0.763

Complications

Patients with asthma were more likely to require an extended length of hospital stay (7.0% vs 3.0%; p=0.020) (Table 3). After controlling for baseline characteristics on multivariate analysis, patients with a history of asthma had an increased risk of extended hospital stay (OR 5.780; 95% CI 1.671 to 19.996; p=0.006) (Table 4). There were no differences in other postoperative complications between the two groups.

**Table 3:** Bivariate Analysis of Postoperative Complications of Patients Following Tibial Shaft Fracture Treatment ¶Pearson’s chi-squared test Bolding equals significance p<0.05

Complications	No Asthma	Asthma	P-value^¶^
Total patients, n	2,534	115	
Superficial surgical site infection, n (%)	14 (0.6)	0 (0.0)	0.424
Deep surgical site infection, n (%)	3 (0.1)	0 (0.0)	0.712
Superficial wound dehiscence, n (%)	4 (0.3)	0 (0.0)	0.647
Deep wound dehiscence, n (%)	4 (0.2)	0 (0.0)	0.670
Pneumonia, n (%)	0 (0.0)	0 (0.0)	-
Urinary tract infection, n (%)	2 (0.1)	0 (0.0)	0.763
Postoperative transfusion, n (%)	6 (0.2)	0 (0.0)	0.601
Venous thromboembolism, n (%)	2 (0.1)	0 (0.0)	0.763
Sepsis, n (%)	1 (0.0)	0 (0.0)	0.831
Extended length of stay (> 5 days), n (%)	77 (3.0)	8 (7.0)	0.020
Reoperation, n (%)	36 (2.5)	2 (2.6)	0.941
Readmission, n (%)	24 (1.6)	3 (3.9)	0.142
Death, n (%)	0 (0.0)	0 (0.0)	-

**Table 4:** Multivariate Analysis of Postoperative Complications of Patients Following Tibial Shaft Fracture Treatment Bolding equals significance p<0.05 CI, confidence interval.

Complications	Asthma	
	p-value	Odds ratio (yes asthma/no asthma) (95% CI)
Extended length of stay (> 5 days)	0.006	5.780 (1.671 to 19.996)

## Discussion

Given the high prevalence of both tibial shaft fractures and asthma in the pediatric population, understanding the potential implications of asthma on the surgical management of tibial shaft fractures may improve postoperative treatment courses. This study sought to determine the association between asthma and 30-day postoperative complications for tibial shaft fractures. Overall, patients with asthma did not experience high rates of postoperative complications, however, they were found to have an increased length of hospital stay compared to their non-asthmatic counterparts.

Asthma has a wide range of severity and chronicity, involving varying degrees of airway inflammation, hyperresponsiveness, degree of obstruction, and symptomatology [13]. An asthmatic patient undergoing surgery is therefore at an increased risk for perioperative morbidity and mortality. Detailed patient history and preoperative assessment, including pulmonary function testing, are crucial to reduce the potential risk of surgical complications [10,11,13]. Due to the increased airway hyperreactivity observed in asthmatics, bronchospasm may readily be precipitated by instrumentation, therapeutic drugs, and perioperative complications such as aspiration, infection, or trauma [11,13]. Anesthesia-induced asthma exacerbations can also be caused by anesthesia's effect on diaphragmatic function, the ability to cough, and the function of the mucociliary escalator [11,13]. As a result, these identified alterations in lung function may lead to postoperative atelectasis, mucus plugging, and wheezing, thus requiring increased medical attention [13]. Additionally, emergence from anesthesia presents a risk of laryngospasm and bronchospasm [13]. All of these potential complications can precipitate the need for a longer hospital course.

Although the literature demonstrates an increased risk for perioperative complications in patients with asthma [10,11,13], only a few studies specifically investigate postoperative outcomes in this patient population. A database created by Benyo et al. looking at risk factors and postoperative complications following septoplasty in pediatric patients found that a history of asthma was found to be a significant risk factor for increased postoperative complications (p=0.028) [14]. However, no association between a diagnosis of asthma and extended hospital stay was found for children undergoing septoplasty [14], whereas our study found a significant increase in length of stay among children with asthma following surgical repair of tibial shaft fractures.

There are a number of factors that may contribute to the length of hospital stay following tibial fracture surgery in pediatric patients. The most common and challenging complications following open fractures include infection, bone-healing complications, such as delayed union and non-union, and compartment syndrome [15,16]. Infection after open fractures occur in 2%-10% of patients and can be treated with local wound care and oral antibiotic therapy. However, progression to deep soft-tissue infection and osteomyelitis may occur and require further surgical irrigation and debridement [15,17]. Delayed union and non-union are less common but if they do occur, the surgeon must consider possible deep infection, hardware failure, or fracture site instability [15]. Our study did not show an increased rate of any of these complications among patients who presented with a diagnosis of asthma. Further retrospective and prospective studies would be needed to evaluate the role of asthma in precipitating the above complications.

The results of this study should be viewed in the context of its strengths and limitations. One strength of this study is that NSQIP is a large patient database that allowed us to compile detailed patient information from across the nation and obtain a large, varied sample size with statistical power. However, this study has several limitations as well. Data from the NSQIP database are collected from select participating institutions, which may not be representative of the entire pediatric population with asthma requiring surgical management for tibial shaft fractures. Additionally, there is potential for coding errors with regard to diagnosis, complications, and comorbidities. Finally, the dataset is limited to 30-days postoperative outcomes, which does not capture complications that occur beyond this period, limiting our ability to investigate intermediate and long-term outcomes. Furthermore, we did not stratify the patients by the type of surgical repair that was done to treat the tibial shaft fracture. This type of stratification can be included in future studies on this topic.

## Conclusions

In conclusion, this study analyzed the impact of asthma on the incidence of 30-day postoperative complications and length of stay following the surgical management of tibial shaft fractures in pediatric patients. Our results indicated that pediatric patients with asthma had an increased risk of prolonged hospital stay following surgery compared to those without asthma. However, no significant differences in the 30-day postoperative complications assessed in this study were seen between the two cohorts. This study begins to add to the growing literature investigating outcomes in pediatric patients diagnosed with asthma. Prior studies have shown that asthma is an independent risk factor for complications following surgery, however, additional research is necessary to determine how complications associated with asthma impact specific operative outcomes. Adequate training and awareness around this association are critical for pediatric care teams to improve the quality of patient care while reducing patient stress and the associated healthcare costs resulting from longer hospital stays.
